# Testing a beverage-context explanation for caffeinated beverage consumption motives in Japan using beverage-specific questionnaire framing

**DOI:** 10.3389/fnut.2026.1888996

**Published:** 2026-08-17

**Authors:** Naoki Kato, Shunji Oshima, Katsumi Watanabe

**Affiliations:** 1 Core Technology Laboratories, Asahi Quality & Innovations, Ltd., Ibaraki, Japan; 2 Faculty of Science and Engineering, Waseda University, Tokyo, Japan; 3 Sustainable Technology Laboratories, Asahi Quality & Innovations, Ltd., Ibaraki, Japan

**Keywords:** caffeine consumption, caffeine consumption motives, green tea, Japan, measurement invariance, Motives for Caffeine Consumption Questionnaire

## Abstract

**Background:**

Motives for consuming caffeinated beverages may vary by beverage type, yet it remains unclear whether the suboptimal fit of the provisional Japanese version of the Motives for Caffeine Consumption Questionnaire (MCCQ-J) reflects beverage-specific contexts, such as green tea, in Japan. This study compared all-beverage vs. beverage-specific responding on the MCCQ-J and profiled beverage-wise motives.

**Methods:**

Two online surveys were conducted using a provisionally translated Japanese version of the MCCQ (MCCQ-J), prepared through forward–backward translation without full linguistic validation. In Survey 1, the MCCQ-J was completed by 1,530 individuals (20–59 years) considering all caffeinated beverages (all-beverage framing). In Survey 2, 1,190 follow-up participants who consumed at least one of the four target beverages completed the MCCQ-J under beverage-specific framing, limiting responses to a single beverage type. Six subscale scores (habit, alertness, mood, social, taste, and symptom management) were calculated as in the original study, and six-factor model fit, internal consistency, and measurement invariance across beverage types were evaluated.

**Results:**

The six-factor model fit was suboptimal under all-beverage framing and did not uniformly improve under beverage-specific framing. Invariance patterns across beverage pairs were not specific to green tea, making the findings inconsistent with a green-tea-specific explanation of misfit. In the four-group beverage-type comparison, full scalar invariance was unsupported, and acceptable partial scalar invariance could not be established. Therefore, beverage-type score differences were interpreted only descriptively. Mood motives appeared descriptively more prominent than in the original MCCQ study, but this observation should be interpreted cautiously because cross-national invariance was not tested.

**Conclusions:**

Evidence for beverage-type comparability under the provisional MCCQ-J framework is limited, and the data are inconsistent with a green-tea-specific explanation of misfit. The findings offer descriptive references for Japanese caffeine-consumption motives and highlight the need for further refinement of the MCCQ-J (e.g., cognitive interviews, item rewording, pilot testing).

## 1 Introduction

Today, caffeinated beverages are consumed worldwide, and there are differences in the market composition of caffeinated beverages across countries (1). Beverages such as coffee, tea, and energy drinks differ not only in caffeine content but also in sensory characteristics, potential social meanings, and typical consumption contexts, which may lead to variation in motives for caffeine consumption across beverage types. Consequently, differences in the market composition of caffeinated beverages across countries may be associated with corresponding differences in caffeine-consumption motives. To examine such motives systematically, psychological scales are needed. However, in Japan, where this study was conducted, a fully validated Japanese version of a global caffeine-motive scale has not yet been established, limiting the ability to examine caffeine-consumption motives in relation to the composition of caffeinated-beverage markets in Japan.

Researchers have developed psychological scales to measure the motives for caffeine consumption, and the Caffeine Motives Questionnaire (CMQ) and the Motives for Caffeine Consumption Questionnaire (MCCQ) are well known (2, 3). The CMQ has not been widely adapted into languages other than English, while the MCCQ has been adapted into Turkish in addition to English (4). No other cross-cultural adaptations of the MCCQ have been reported to date. The CMQ and MCCQ have different factor structures, with the CMQ having a four-factor structure and the MCCQ having a six-factor structure. Specifically, the MCCQ is composed of habit, alertness, mood, social, taste, and symptom management. While the original MCCQ instructions ask participants to think about all caffeinated beverages they consume, not just certain types, Ágoston et al. (2) reported that these subscale scores may differ by participants' primary source of caffeine intake (e.g., coffee vs. tea). These patterns suggest a possible association between cross-country market composition of caffeinated beverages and the MCCQ subscale means.

The factor structure can remain stable even when motive means differ across countries. Notably, the Turkish version replicated the original six-factor structure with excellent fit, suggesting the potential cross-cultural applicability of the six-factor framework (4). On the other hand, when comparing motive levels across groups (e.g., gender or age) or across beverage types, establishing measurement invariance is important to ensure that observed differences reflect substantive differences rather than measurement artifacts.

Against this background, in a prior paper based on Survey 1 data from the same broader research project but addressing a different research question, we provisionally adapted the Japanese version of the MCCQ (MCCQ-J) through forward-backward translation and observed poorer model fit for the six-factor model under all-beverage responding relative to the original study (2, 5). We hypothesized that the original all-beverage framing may prompt respondents to recall different beverages when answering, which could lead to poor model fit. *Ryoku-cha* (green tea), which is more common in Japan than in Hungary, may be one of the contextual factors contributing to poorer model fit of the six-factor structure in the MCCQ-J. In Japan, green tea is associated with hospitality, mealtime pairing, and mood adjustment, and it retains culturally specific meanings that differ from typical coffeehouse or energy drink contexts (6). Such context variation may introduce heterogeneity in how respondents interpret and respond to general questionnaire items, potentially affecting the comparability of item function across respondents. For example, motives related to mood regulation may be interpreted differently depending on whether respondents recall beverages typically consumed in solitary vs. socially embedded contexts. Because this hypothesis was exploratory, we did not assume that green tea would affect only a single MCCQ factor; rather, context-dependent motives such as mood, social, habit, and taste motives were considered potentially susceptible to beverage-specific interpretation. Importantly, such effects are not specific to green tea, and similar heterogeneity may arise for other culturally embedded beverages. In addition, existing evidence on green tea mainly concerns reasons for drinking green tea itself and does not directly show that green tea alters responses to a general caffeine-consumption motive questionnaire. Therefore, the present study treated the green-tea context as an exploratory hypothesis rather than as an established explanation for MCCQ-J model misfit.

Accordingly, this study had two aims: (1) to examine whether the suboptimal fit under all-beverage framing was consistent with a *ryoku-cha* (green tea)-specific context explanation by comparing all-beverage and beverage-specific framings and evaluating measurement invariance (beverage-specific framing = instructing respondents to answer all MCCQ-J items with one named beverage—coffee, *ryoku-cha* [green tea], *kou-cha* [black tea], or energy drink; all-beverage framing = considering all caffeinated beverages consumed); and (2) to descriptively explore beverage-wise score patterns among Japanese participants under the original six-factor MCCQ framework.

If the green-tea-specific explanation were supported, poorer model fit and measurement non-invariance would be expected to be most evident in the *ryoku-cha* condition or in beverage pairs involving *ryoku-cha*. Conversely, if similar misfit and non-invariance were also observed in non-green-tea conditions or pairs, this would be inconsistent with a uniquely green-tea-specific explanation, although it would not rule out the possibility that green tea contributes to response heterogeneity. Specifically, whereas the prior paper focused on associations involving motives for alcohol and caffeine consumption using Survey 1 data, the present study administered the MCCQ-J under a beverage-specific framing in Survey 2, contrasted these responses with the all-beverage framing, and examined beverage-type comparability and descriptive score patterns. Participants also self-reported the beverage that first comes to mind when thinking about caffeinated beverages. This allowed us to approximate which beverage participants may primarily have had in mind when answering the MCCQ-J under the all-beverage framing. This study provides an initial exploration of beverage-specific caffeine-consumption motive profiles among Japanese participants using the MCCQ framework, a motive scale that has been used in multiple countries, and will contribute to understanding how people have interacted with caffeinated beverages within their respective cultures.

## 2 Materials and methods

### 2.1 Study design and participants

We conducted two online surveys with funding from Asahi Quality & Innovations, Ltd (AQI). Survey 1 collected the MCCQ-J responses under all-beverage framing, whereas Survey 2 collected the MCCQ-J responses under beverage-specific framing. In both surveys, the participants were recruited from the survey panels of Cross Marketing Group Inc., a major Internet survey company in Japan. Although part of Survey 1 overlaps with the dataset used in the prior paper (5), the present study differs in its research objective, analytic sample definition, and use of follow-up Survey 2 data. The recruitment period for Survey 1 spanned from July 9, 2024, to July 31, 2024, and that for Survey 2 spanned from November 1, 2024, to November 11, 2024.

First, the participants for Survey 1 were briefed about the objective and procedure of the study, and informed consent was obtained online (this procedure was the same in Survey 2). They then completed the demographic survey, which requested the following information: gender, age, marriage, cohabitants/cohabiting families, occupation, and occupation of family members. Participants were excluded if they or their cohabiting family members worked in the food & beverage, food service, mass media, or marketing research industries. These exclusions were predefined to reduce the risk that occupational expertise or industry attitudes might introduce systematic bias in responses when completing the questionnaire. The sample size for Survey 1 was determined based on AQI's budget, and a total of 1,530 participants in Survey 1, aged between 20 and 59 years, responded to the questionnaires described in Section 2.3 (*M*_*age*_ = 40.55, *SD* = 9.96; female: 56.27%; age range/distribution: 20s: 15.88%, 30s: 33.59%, 40s: 28.10%, 50s: 22.41%). Survey 1 was originally designed as a broader beverage-related survey that also included alcohol-related measures. Accordingly, the target age range for Survey 1 was set at 20–59 years, reflecting the legal drinking age in Japan and the age range adopted in prior Japanese alcohol-motive research (7). For Survey 2, 1,313 participants were recruited by recontacting participants from Survey 1 and responded to the questionnaires described in Section 2.4 (*M*_*age*_ = 41.21, *SD* = 9.87; female: 56.66%; age range/distribution: 20s: 14.09%, 30s: 32.74%, 40s: 28.94%, 50s: 24.22%). To examine potential attrition bias between Survey 1 and Survey 2, baseline characteristics measured in Survey 1 were compared between non-completers (*n* = 217) and completers (*n* = 1,313). Categorical characteristics are summarized in Supplementary Table S1, and continuous variables are summarized in Supplementary Table S2.

Specifically, the analytic sample was limited to respondents who consumed caffeinated beverages at least 1 day in the past 30 days. In the prior paper based on this project (5), the analytic sample was further restricted to respondents in their 20s and 30s who were screened for low-risk drinking, in accordance with that paper's objective of examining associations between motives for alcohol and caffeine consumption while reducing heterogeneity related to higher-risk drinking patterns and facilitating age comparability with previous MCCQ studies. In contrast, the present study addressed a different question—namely, the psychometric properties of the MCCQ-J and beverage-specific score patterns in a broader adult sample—and therefore did not apply those additional age- and alcohol-risk-based analytic restrictions. This approach also allowed closer comparison with the original MCCQ study (2), which does not impose participant conditions regarding alcohol drinking behavior. As a result, data analysis for this study was conducted on 1,207 people (*M*_*age*_ = 41.67, *SD* = 9.75; female: 56.17%; age group/distribution: 20s: 12.59%, 30s: 32.23%, 40s: 29.99%, 50s: 25.19%). Among these participants, 1,190 reported consuming at least one of the four target beverages and were assigned to one beverage-specific MCCQ-J condition; the remaining 17 participants were not included in beverage-specific CFA or measurement invariance analyses.

The participants who completed all items were sent an Amazon gift certificate worth 2,000 yen as a reward for each of Survey 1 and Survey 2. The Ethics Committee of User Life Science, Ltd. approved the protocol of the study (reference numbers: ULS 202406 for Survey 1 and ULS 202410 for Survey 2).

### 2.2 Translation and provisional adaptation of the MCCQ-J

With permission from the original authors, we provisionally adapted the MCCQ into Japanese via forward-backward translation. Translation and back-translation were outsourced to a contractor, and we verified the results. Specifically, two native Japanese translators independently translated the original MCCQ into Japanese, and the two forward translations were combined into a single version. This version was then back-translated by a native English translator. The back-translation was reviewed by a native English reviewer against the original version. No major differences in meaning were identified, and conceptual equivalence was provisionally judged to be acceptable based on the translation and back-translation review by the translators and authors. To increase transparency regarding conceptual equivalence, the original items, forward translation, back-translation, and provisional conceptual equivalence notes are provided in Supplementary Table S3. Although no cognitive interviews, expert panel review, or pilot testing were conducted prior to the present study, the translated instrument was examined here as a provisional Japanese adaptation.

### 2.3 Measures in Survey 1

#### 2.3.1 Frequency of caffeinated beverage consumption

Participants self-reported, for caffeinated beverages, (a) the total number of days consumed in the past 30 days (overall days) and (b) the number of days consumed alone (solitary days). We additionally derived a “with-others days” variable as overall minus solitary (values below zero, if any, were set to zero). No negative values were observed.

#### 2.3.2 Japanese version of the Motives for Caffeine Consumption Questionnaire (MCCQ-J)

To measure motives for consuming caffeinated beverages, the participants completed the Japanese version of the MCCQ (2). The original version consists of 23 items with six subscales, namely, habit, alertness, mood, social, taste, and symptom management. The items were rated using a five-point Likert scale (from 1 = *Never*/*Almost never* to 5 = *Almost every time*/Always). The participants who did not typically consume caffeinated beverages were asked to select 1 = *Never*/*Almost never*.

### 2.4 Measures in Survey 2 and beverage-specific framing

#### 2.4.1 The beverage that first comes to mind when thinking about caffeinated beverages

The participants self-reported the beverage that first comes to mind when thinking about caffeinated beverages in response to the following question: “Among the beverages you drink regularly, which of the following best describes a caffeinated beverage? Please select the one that best applies.” The options were “coffee,” “*ryoku-cha* (green tea),” “*kou-cha* (black tea),” “energy drink,” “the others” and “I don't drink anything with caffeine.”

#### 2.4.2 Regular consumption of each caffeinated beverage

The participants answered the following questions regarding their usual consumption of coffee, *ryoku-cha* (green tea), *kou-cha* (black tea), and energy drink: “Do you drink or eat any of the following beverages or foods? Please indicate all that apply.” The options were “drink/eat it” and “never drink/eat it.” Although it was outside the scope of the study, the reason for asking about this tendency through the description “Do you drink or eat any of the following beverages or foods?” was that the participants were also asked about the contents of other food and beverages such as beer and chocolate in the online survey.

#### 2.4.3 Frequency of consumption for each caffeinated beverage

The participants who answered “drink/eat it” to the question in Section 2.4.2. regarding coffee, *ryoku-cha* (green tea), *kou-cha* (black tea), and energy drink self-reported, for each beverage type, the total number of days consumed in the past 30 days. The participants who answered “never drink/eat it” to the question in Section 2.4.2 were assigned a score of 0.

#### 2.4.4 The motives for each caffeinated beverage consumption (MCCQ-J under beverage-specific framing)

Variation in the MCCQ-J factor structure and subscale profiles by beverage type was examined to address the poor fit under all-beverage framing observed in Survey 1. Accordingly, the participants who answered “drink/eat it” in Section 2.4.2. for at least one of coffee, *ryoku-cha* (green tea), *kou-cha* (black tea), and energy drink were asked about their motives for drinking one of the caffeinated beverages they answered “drink/eat it” for. Specifically, whereas the original MCCQ instructs respondents to consider all caffeinated beverages they consume rather than any single type, we added a beverage-specific framing and asked participants to answer all 23 MCCQ-J items with the instruction: “Please answer the following questions about X (X = coffee, *ryoku-cha* (green tea), *kou-cha* (black tea), or energy drink).” The beverage prompts were assigned with the aim of achieving as balanced a distribution across beverage types as possible among the beverages each participant reported consuming. Participants who reported consuming multiple beverages were dynamically assigned to one beverage condition. Specifically, participants were assigned to the beverage with the lowest number of responses at the time of participation among the eligible beverage options. If more than one eligible beverage had the same lowest response count, one of those beverages was selected randomly by the survey system. This allocation procedure was pre-specified prior to data collection and implemented through the survey platform. However, the allocation algorithm was not formally preregistered. Therefore, beverage assignment depended on participants' reported consumption of the four target beverages and was not independent of beverage-use patterns. This beverage-specific framing served the primary test of beverage-context effects.

### 2.5 Statistical analysis

Data were analyzed using IBM SPSS Statistics version 29.0.1 and Amos version 29.0. We reported two-sided *p-values* and effect sizes. To control for multiple testing across the many correlational and group-comparison tests, we applied the Benjamini–Hochberg false discovery rate (BH-FDR) procedure within each table (*q* < 0.05). Results surviving BH-FDR correction are indicated in the tables (bold). The interpretation of the absolute value of the effect size followed the classification of Mizumoto and Takeuchi (8), using |*r*| ≥ 0.10 for correlations and |*d*| ≥ 0.20 for mean differences as indicative benchmarks rather than strict cut-offs, as these thresholds were suggested by Mizumoto and Takeuchi as reference values. Because even small effects can become statistically significant in large samples, we interpreted findings by considering statistical significance, BH-FDR correction, and effect size magnitude together, and avoided treating small effects as substantively important solely because they survived correction. Findings with small effect sizes were interpreted cautiously, even when statistically significant after BH-FDR correction.

To examine whether the distribution of caffeinated beverages differed by gender and age group for (a) the beverage that first comes to mind when thinking about caffeinated beverages (see Section 2.4.1) and (b) the beverage assignment based on “drink/eat it” responses under the MCCQ-J's beverage-specific framing (see Section 2.4.4 and Supplementary Table S4), the Chi-square test was used. In addition, attrition from Survey 1 to Survey 2 was examined by comparing non-completers and completers on baseline categorical variables using the Chi-square tests (Supplementary Table S1).

Gender differences in the items/subscale scores described in Sections 2.3 and 2.4 were assessed using Welch's *t*-test. Baseline continuous differences between non-completers and completers were also assessed using Welch's *t*-tests (Supplementary Table S2).

For comparisons between the subscale scores of the MCCQ-J for each caffeinated beverage, one-way ANOVAs were conducted. For Supplementary Table S5, BH-FDR correction was applied to the omnibus ANOVA *p*-*values* across subscales, and Games-Howell *post-hoc* pairwise comparisons were reported only for subscales whose omnibus tests remained significant after correction (*q* < 0.05).

Confirmatory factor analyses (CFA) were conducted to evaluate the MCCQ-J under the original six-factor structure (habit, alertness, mood, social, taste, symptom management). The reported indices were χ^2^, CFI, TLI, RMSEA, and SRMR, and their interpretation referenced Hu and Bentler (9), using benchmarks close to 0.95 for CFI/TLI, 0.08 for SRMR, and 0.06 for RMSEA. Internal consistency was assessed using Cronbach's alpha. For subscales consisting of two items, inter-item correlations were also examined and reported as a complementary index of internal consistency. To provide item-level information regarding model misfit, modification indices and standardized residual covariances from the six-factor CFA under all-beverage framing were also examined.

We tested measurement invariance hierarchically (configural → metric → scalar). Measurement invariance was primarily evaluated using ΔCFI ( ≤ 0.010) and ΔRMSEA ( ≤ 0.015) (10, 11), with ΔSRMR also examined as supplementary evidence because recommended SRMR cutoff values vary across studies. For reference, ΔSRMR ≤ 0.030 for metric invariance and ≤ 0.015 for scalar invariance were used in the present study. Specifically, invariance was examined (a) across gender and age groups under all-beverage framing and (b) across beverage types under beverage-specific framing. To directly probe the *ryoku-cha* (green tea) context hypothesis, we additionally conducted two-group CFAs for beverage pairs, contrasting pairs that included green tea with pairs that did not. When full scalar invariance was not supported, partial scalar invariance was examined by sequentially freeing intercept constraints guided by modification indices, while retaining at least two anchor items per factor. The adequacy of the partial scalar model was evaluated by comparing it with the metric invariance model using the same ΔCFI, ΔRMSEA, and ΔSRMR criteria applied to scalar invariance. Latent mean comparisons across beverage types were planned only if full or acceptable partial scalar invariance was supported.

The relationship among age, frequency of caffeinated beverage consumption, and the subscale scores of the MCCQ-J were examined using Pearson correlations.

## 3 Results

### 3.1 Attrition analysis from Survey 1 to Survey 2

The final analytic sample consisted of 1,207 participants aged 20–59 years (*Mage* = 41.67, *SD* = 9.75; female: 56.17%). Of these, 1,190 participants were included in the beverage-specific CFA and measurement-invariance analyses.

To examine potential attrition bias, baseline characteristics measured in Survey 1 were compared between non-completers of Survey 2 (*n* = 217) and completers of Survey 2 (*n* = 1,313). As a result, age-group distribution differed between the two groups, whereas gender and the proportion of participants who had consumed caffeinated beverages at least 1 day in the past 30 days did not show clear differences (Supplementary Table S1). Given that age-group differences were observed, the Survey 2 sample may not fully represent the age composition of the original Survey 1 sample, and beverage-specific CFA and measurement-invariance results should therefore be interpreted with this possible attrition-related age shift in mind. Additionally, none of the baseline continuous behavioral or MCCQ-J subscale variables remained significant after BH-FDR correction (Supplementary Table S2). Furthermore, none of the effect sizes exceeded the indicative benchmark used in this study. Therefore, although age-group differences were observed, evidence for meaningful baseline differences in the measured behavioral variables and MCCQ-J motive scores was limited.

### 3.2 Gender and age group differences in the beverage first associated with caffeine and assigned beverage type

Table 1 shows the distribution of the beverage that first comes to mind when thinking about caffeinated beverages by gender and age group. For the comparison by gender, the results of the Chi-squared test were χ^2^_(5)_ = 44.06, *p* < 0.001, and Cramer's *V* = 0.19. Approximately 80% of both males and females chose coffee, but females showed higher selection rates for *ryoku-cha* (green tea) and *kou-cha* (black tea) and a lower selection rate for energy drink than males. Additionally, for the comparison across age groups, the Chi-squared test yielded χ^2^_(15)_ = 86.50, *p* < 0.001, and Cramer's *V* = 0.16. Coffee accounted for more than 50% of choices in all age groups, but the younger the age group, the lower the percentage of coffee choices, while *ryoku-cha* (green tea), *kou-cha* (black tea), and energy drinks showed the opposite trend.

**Table 1:** Gender and age group difference in the beverage that first comes to mind when thinking about caffeinated beverages.

		***N*** (%)							*χ^2^* and Cramer's *V*
Characteristic	Category	Total	Coffee	*Ryoku-cha* (green tea)	*Kou-cha* (black tea)	Energy drink	The others	I don't drink anything with caffeine	
**Total**		1,207 (100.00)	969 (80.28)	72 (5.97)	57 (4.72)	76 (6.30)	14 (1.16)	19 (1.57)	
Gender	Male	529 (43.83)	432 (81.66)	26 (4.91)	8 (1.51)	52 (9.83)	3 (0.57)	8 (1.51)	*χ^2^*_(5)_ = 44.06
	Female	678 (56.17)	537 (79.20)	46 (6.78)	49 (7.23)	24 (3.54)	11 (1.62)	11 (1.62)	Cramer's *V* = 0.19^**^
Age group	20–29 years (57.24% Female)	152 (12.59)	88 (57.89)	19 (12.50)	11 (7.24)	20 (13.16)	3 (1.97)	11 (7.24)	*χ^2^*_(15)_ = 86.50
	30–39 years (51.67% Female)	389 (32.23)	307 (78.92)	24 (6.17)	22 (5.66)	29 (7.46)	5 (1.29)	2 (0.51)	Cramer's *V* = 0.16^**^
	40–49 years (54.42% Female)	362 (29.99)	310 (85.64)	14 (3.87)	14 (3.87)	17 (4.70)	3 (0.83)	4 (1.10)	
	50–59 years (63.49% Female)	304 (25.19)	264 (86.84)	15 (4.93)	10 (3.29)	10 (3.29)	3 (0.99)	2 (0.66)	

Gender difference in age group was assessed using the Chi-square test: *p* = 0.016, χ^2^_(3)_ = 10.33, Cramer's *V* = 0.09.

The response option “I don't drink anything with caffeine” referred to participants' self-identified regular beverages in Survey 2 and did not necessarily contradict the analytic-sample criterion of having consumed caffeinated beverages at least one day in the past 30 days.

Percentages in the Total column indicate proportions of the entire sample, whereas percentages in the other columns indicate proportions within each subgroup.

Given the limited number of planned comparisons, *p*-values were not adjusted for multiple testing.

^**^p < 0.01.

Under beverage-specific framing, participants were assigned to respond with a single beverage they answered “drink/eat it” for in Section 2.4.2, with the aim of keeping beverage-group sizes as balanced as possible. Supplementary Table S4 shows that the distribution of assigned beverage type differed by gender [χ^2^_(3)_ = 42.73, *p* < 0.001, and Cramer's *V* = 0.19] and age group [χ^2^_(9)_ = 24.78, *p* < 0.001, and Cramer's *V* = 0.08]. Thus, descriptive beverage-type differences should be interpreted with caution because assigned beverage groups differed in demographic composition.

### 3.3 Confirmatory factor analysis and internal consistency of the six-factor structure of MCCQ-J

Table 2 shows the results of each CFA of the fit indices in the same six-factor structure of the MCCQ-J as the original English version under all-beverage framing. In contrast to the original study, under all-beverage framing, only the SRMR met the benchmark value for model fit [χ^2^ = 3,112.35 (*df* = 215, *p* < 0.001]), CFI = 0.864, TLI = 0.840, RMSEA = 0.106, SRMR = 0.075]. Model fit was broadly similar across gender and age groups, although fit tended to be relatively poorer among females [χ^2^ = 1,897.83 (*df* = 215, *p* < 0.001), CFI = 0.859, TLI = 0.834, RMSEA = 0.108, SRMR = 0.081] and in older age groups [50s: χ^2^ = 1,035.71 (*df* = 215, *p* < 0.001), CFI = 0.847, TLI = 0.820, RMSEA = 0.112, SRMR = 0.081]. Regarding item-level misfit under all-beverage framing, several alertness-related items showed relatively large modification indices and standardized residual covariances (Supplementary Table S6). In addition, item 13 (“because I become refreshed”) showed a notable potential cross-loading on the taste factor, and item 19 (“because it brings me together with other people”) showed a potential cross-loading on the habit factor.

**Table 2:** Fit indices of CFA results of the MCCQ-J under all-beverage framing.

Group	*N*	*χ^2^*	CFI	TLI	RMSEA	SRMR
**Total**	1,207	3,112.35 (*df* = 215, *p* < 0.001)	0.864	0.840	0.106	0.075
Male	529	1,439.61 (*df* = 215, *p* < 0.001)	0.873	0.850	0.104	0.070
Female	678	1,897.83 (*df* = 215, *p* < 0.001)	0.859	0.834	0.108	0.081
20–29 years (57.24% Female)	152	661.61 (*df* = 215, *p* < 0.001)	0.853	0.827	0.117	0.076
30–39 years (51.67% Female)	389	1,183.39 (*df* = 215, *p* < 0.001)	0.861	0.836	0.108	0.080
40–49 years (54.42% Female)	362	1,142.48 (*df* = 215, *p* < . 001)	0.851	0.825	0.109	0.079
50–59 years (63.49% Female)	304	1,035.71 (*df* = 215, *p* < 0.001)	0.847	0.820	0.112	0.081

Participants who consumed caffeinated beverages at least one day in the past 30 days (Section 2.3.1.).

MCCQ-J, Japanese version of the Motives for Caffeine Consumption Questionnaire.

On the other hand, even when the MCCQ-J data were obtained under beverage-specific framing, not all fit indices met the benchmark values for model fit, and the fit indices were particularly poor for coffee [χ^2^ = 1,000.47 (*df* = 215, *p* < 0.001), CFI = 0.870, TLI = 0.846, RMSEA = 0.110, SRMR = 0.081] (Table 3). By contrast, the energy drink condition showed relatively better fit than the other beverage-specific conditions, although RMSEA remained above the recommended benchmark and this pattern should be interpreted cautiously given demographic differences among assigned beverage groups.

**Table 3:** Fit indices of CFA results of the MCCQ-J under beverage-specific framing.

Condition	*N*	*χ^2^*	CFI	TLI	RMSEA	SRMR
Coffee condition	304	1,000.47 (*df* = 215, *p* < 0.001)	0.870	0.846	0.110	0.081
*Ryoku-cha* (green tea) condition	291	905.81 (*df* = 215, *p* < 0.001)	0.889	0.869	0.105	0.065
*Kou-cha* (black tea) condition	292	968.83 (*df* = 215, *p* < 0.001)	0.878	0.856	0.110	0.072
Energy drink condition	303	735.37 (*df* = 215, *p* < 0.001)	0.918	0.904	0.090	0.062

CFA was conducted separately for each beverage-specific framing condition. Participants were assigned to one beverage condition among the beverages they reported consuming.

MCCQ-J, Japanese version of the Motives for Caffeine Consumption Questionnaire.

Under both all-beverage framing and beverage-specific framing, no items with factor loadings of less than 0.5 were found, but the reliability analysis results showed that only the symptom management subscale for coffee and the habit subscale for *ryoku-cha* (green tea) had Cronbach's alpha < 0.70 (Supplementary Tables S7, S8).

### 3.4 Measurement invariance and subscale scores under all-beverage framing

Table 4 shows the results of hierarchical measurement invariance testing (configural → metric → scalar) under all-beverage framing. Scalar invariance was supported across gender (ΔCFI = −0.009, ΔRMSEA = 0.000, ΔSRMR = 0.000) and across age groups (ΔCFI = −0.005, ΔRMSEA = −0.001, ΔSRMR = 0.003), indicating that mean-level comparisons of the MCCQ-J subscales are permissible across these groups under all-beverage framing. However, the ΔCFI value for gender was close to the ΔCFI ≤ 0.010 criterion, indicating that mean-level gender comparisons should be interpreted with caution.

**Table 4:** Hierarchical measurement invariance testing across gender and age groups under all-beverage framing.

Grouping variable	*N*	Group *N*s	Model	CFI	TLI	RMSEA	SRMR	ΔCFI	ΔRMSEA	ΔSRMR	Decision
Gender	1,207	Male = 529; Female = 678	Configural	0.865	0.841	0.075	0.070				
			Metric (loadings)	0.865	0.847	0.074	0.070	0.000	−0.001	−0.000	Invariant
			Scalar (intercepts)	0.856	0.845	0.074	0.070	−0.009	0.000	0.000	Invariant
Age group	1,207	20s = 152; 30s = 389; 40s = 362; 50s = 304	Configural	0.853	0.827	0.055	0.076				
			Metric (loadings)	0.854	0.838	0.054	0.076	0.001	−0.001	0.000	Invariant
			Scalar (intercepts)	0.849	0.844	0.053	0.079	−0.005	−0.001	0.003	Invariant

Decision criteria: ΔCFI ≤ 0.010, ΔRMSEA ≤ 0.015; ΔSRMR ≤ 0.030 (metric) / ≤ 0.015 (scalar).

Supplementary Table S9 shows the average subscale scores of MCCQ-J when data were obtained under all-beverage framing. Specifically, habit mean score was 3.30 (*SD* = 1.23), alertness mean score was 2.66 (*SD* = 1.05), mood mean score was 2.88 (*SD* = 1.27), social mean score was 1.91 (*SD* = 0.99), taste mean score was 3.76 (*SD* = 1.23), and symptom management mean score was 1.59 (*SD* = 0.88).

Regarding gender differences in those subscale scores, BH-FDR-corrected results indicated that females scored higher on taste (*q* < 0.001, *d* = −0.22), whereas males scored slightly higher on symptom management (*q* = 0.042, *d* = 0.14) (Table 5).

**Table 5:** Gender difference in frequency of caffeinated beverage consumption and the subscale scores of the MCCQ-J under all-beverage framing.

Variable	***M*** (***SD***)		*t-*test (*df*)	*p* (uncorrected)	*q* (BH-FDR)	Cohen's *d*	95% CI
	Male (*N* = 529)	Female (*N* = 678)					
Frequency of caffeinated beverage consumption							
Overall days	22.60 (10.05)	23.13 (9.85)	−0.92 (1,123.98)	0.357	0.469	−0.05	[−0.17, 0.06]
Solitary days	21.10 (10.83)	20.79 (10.96)	0.50 (1,140.78)	0.620	0.620	0.03	[−0.09, 0.14]
With-others days	1.50 (4.87)	2.34 (6.20)	−2.65 (1,204.96)	0.008	**0.026**	−0.15	[−0.26, −0.04]
Frequency of consumption for each caffeinated beverage							
Coffee	19.35 (11.85)	19.99 (12.14)	−0.91 (1,147.15)	0.361	0.469	−0.05	[−0.17, 0.06]
*Ryoku-cha* (green tea)	13.28 (11.26)	12.82 (11.62)	0.69 (1,150.75)	0.492	0.581	0.04	[−0.07, 0.15]
*Kou-cha* (black tea)	4.53 (7.33)	7.19 (8.99)	−5.66 (1,202.72)	*p* < 0.001	***q*** **<** **0.001**	−0.32	[−0.43, −0.21]
Energy drink	2.47 (4.76)	0.90 (2.75)	6.79 (795.90)	*p* < 0.001	***q*** **<** **0.001**	0.42	[0.30, 0.53]
MCCQ-J subscale score							
Habit	3.22 (1.19)	3.37 (1.25)	−2.08 (1,159.18)	0.038	0.071	−0.12	[−0.23, −0.01]
Alertness	2.69 (1.02)	2.63 (1.08)	1.03 (1,159.67)	0.305	0.469	0.06	[−0.05, 0.17]
Mood	2.79 (1.22)	2.95 (1.30)	−2.09 (1,165.66)	0.037	0.071	−0.12	[−0.23, −0.01]
Social	1.92 (1.00)	1.89 (0.98)	0.52 (1,125.15)	0.602	0.620	0.03	[−0.08, 0.14]
Taste	3.60 (1.21)	3.88 (1.23)	−3.87 (1,142.17)	*p* < 0.001	***q*** **<** **0.001**	−0.22	[−0.34, −0.11]
Symptom management	1.66 (0.93)	1.53 (0.84)	2.40 (1,077.74)	0.016	**0.042**	0.14	[0.03, 0.25]

1,207 participants who consumed caffeinated beverages at least one day in the past 30 days (Section 2.3.1.).

Bold indicates results surviving BH-FDR correction across tests in this table (*q* < 0.05).

MCCQ-J, Japanese version of the Motives for Caffeine Consumption Questionnaire.

Age also showed correlations with some subscale scores that survived BH–FDR correction (e.g., habit: *r* = 0.20, *q* < 0.05; taste: *r* = 0.15, *q* < 0.05) (Table 6).

**Table 6:** Pearson's correlation coefficients among age, frequency of caffeinated beverage consumption, and the subscale scores of the MCCQ-J.

Variable	*M* (*SD*)	1	2	3	4	5	6	7	8	9	10	11	12	13	14
1 Age	41.67 (9.75)	—													
Frequency of caffeinated beverage consumption															
2 Overall days	22.90 (9.94)	**0.32**	—												
3 Solitary days	20.93 (10.90)	**0.29**	**0.86**	—											
4 With-others days	1.97 (5.67)	−0.01	**0.11**	**−0.43**	—										
Frequency of consumption for each caffeinated beverage															
5 Coffee	19.71 (12.01)	**0.34**	**0.61**	**0.55**	0.01	—									
6 *Ryoku-cha* (green tea)	13.02 (11.46)	**0.18**	**0.22**	**0.19**	0.02	**0.14**	—								
7 *Kou-cha* (black tea)	6.02 (8.41)	**0.08**	**0.14**	**0.14**	−0.01	0.03	**0.23**	—							
8 Energy drink	1.59 (3.85)	−0.05	0.01	0.04	−0.05	**0.09**	**0.17**	**0.17**	—						
MCCQ-J															
9 Habit	3.30 (1.23)	**0.20**	**0.44**	**0.38**	0.04	**0.47**	**0.11**	**0.07**	**0.07**	—					
10 Alertness	2.66 (1.05)	0.03	**0.19**	**0.20**	−0.05	**0.33**	**0.10**	0.06	**0.21**	**0.57**	—				
11 Mood	2.88 (1.27)	**0.08**	**0.21**	**0.21**	−0.04	**0.33**	**0.09**	0.03	**0.11**	**0.65**	**0.78**	—			
12 Social	1.91 (0.99)	0.00	**0.06**	0.05	0.02	**0.18**	**0.13**	**0.10**	**0.18**	**0.35**	**0.63**	**0.51**	—		
13 Taste	3.76 (1.23)	**0.15**	**0.30**	**0.28**	0.00	**0.28**	**0.10**	**0.08**	0.03	**0.71**	**0.44**	**0.57**	**0.21**	—	
14 Symptom management	1.59 (0.88)	**−0.06**	0.06	0.06	0.00	**0.13**	**0.10**	**0.09**	**0.19**	**0.22**	**0.50**	**0.36**	**0.65**	**0.07**	—

1,207 participants who consumed caffeinated beverages at least 1day in the past 30 days (Section 2.3.1.).

MCCQ-J, Japanese version of the Motives for Caffeine Consumption Questionnaire. Bold indicates results surviving BH-FDR correction within this table (*q* < 0.05).

Because scalar invariance was supported across gender and age, these gender- and age-related differences are less likely to reflect measurement artifacts within this sample. These findings should be interpreted with caution, as some effects that survived BH-FDR correction were small in magnitude and the overall fit of the six-factor solution was suboptimal. For example, although the correlation between age and habit or taste suggests that older participants tended to report slightly stronger habitual and taste-related motives for caffeinated beverage consumption, the effect sizes were small and should not be overinterpreted.

### 3.5 Measurement invariance and descriptive beverage-type comparisons under beverage-specific framing

Table 7 shows the results of hierarchical measurement invariance testing (configural → metric → scalar) under beverage-specific framing. First, the four-group full scalar invariance was not supported (ΔCFI = −0.029, ΔRMSEA = 0.004, ΔSRMR = 0.003). We then examined partial scalar invariance by sequentially freeing intercept constraints guided by modification indices while retaining at least two invariant intercepts per factor. However, even after freeing 9 intercept constraints, the partial scalar model did not satisfy the prespecified ΔCFI criterion when compared with the metric model. Therefore, scalar or acceptable partial scalar invariance across the four beverage types was not established. Second, in two-group CFAs for beverage pairs, scalar invariance was supported for pairs including green tea (coffee vs. *ryoku*-*cha*; *ryoku*-*cha* vs. *kou*-*cha*), but not supported for *ryoku*-*cha* vs. energy drink. Additionally, scalar invariance was also not supported for pairs that did not include green tea (coffee vs. *kou*-*cha*; coffee vs. energy drink; *kou*-*cha* vs. energy drink).

**Table 7:** Hierarchical measurement invariance testing across beverage types under beverage-specific framing.

Comparison	*N*	Group *N*s	Model	CFI	TLI	RMSEA	SRMR	ΔCFI	ΔRMSEA	ΔSRMR	Decision
Four-group CFA	1190	Coffee = 304; *Ryoku*-*cha* (green tea) = 291; *Kou*-*cha* (black tea) = 292; Energy drink = 303	Configural	0.889	0.869	0.052	0.081				
			Metric (loadings)	0.888	0.876	0.051	0.082	−0.001	−0.001	0.001	Invariant
			Scalar (intercepts)	0.859	0.854	0.055	0.085	−0.029	0.004	0.003	Not invariant
			Partial scalar, 9 intercepts freed	0.875	0.868	0.052	0.083	−0.013	0.001	0.001	Not invariant
Two-group CFA	596	Coffee = 304; *Kou*-*cha* (black tea) = 292	Configural	0.874	0.852	0.078	0.081				
			Metric (loadings)	0.874	0.857	0.076	0.083	0.000	−0.002	0.001	Invariant
			Scalar (intercepts)	0.862	0.851	0.078	0.083	−0.012	0.002	0.001	Not invariant
Two-group CFA	608	Coffee = 304; Energy drink = 303	Configural	0.895	0.876	0.071	0.081				
			Metric (loadings)	0.895	0.881	0.069	0.081	0.000	−0.002	−0.000	Invariant
			Scalar (intercepts)	0.862	0.851	0.077	0.084	−0.033	0.008	0.003	Not invariant
Two-group CFA	595	*Kou*-*cha* (black tea) = 292; Energy drink = 303	Configural	0.898	0.880	0.071	0.072				
			Metric (loadings)	0.897	0.884	0.070	0.073	−0.001	−0.001	0.001	Invariant
			Scalar (intercepts)	0.866	0.856	0.078	0.081	−0.031	0.008	0.008	Not invariant
Two-group CFA	595	Coffee = 304; *Ryoku*-*cha* (green tea) = 291	Configural	0.879	0.858	0.076	0.081				
			Metric (loadings)	0.879	0.863	0.075	0.081	0.000	−0.001	0.000	Invariant
			Scalar (intercepts)	0.869	0.859	0.076	0.083	−0.010	0.001	0.002	Invariant
Two-group CFA	583	*Ryoku*-*cha* (green tea) = 291; *Kou*-*cha* (black tea) = 292	Configural	0.883	0.863	0.076	0.065				
			Metric (loadings)	0.883	0.868	0.075	0.069	0.000	−0.001	0.003	Invariant
			Scalar (intercepts)	0.878	0.869	0.074	0.068	−0.005	−0.001	−0.001	Invariant
Two-group CFA	594	*Ryoku*-*cha* (green tea) = 291; Energy drink = 303	Configural	0.904	0.887	0.069	0.065				
			Metric (loadings)	0.903	0.890	0.068	0.066	−0.001	−0.001	0.001	Invariant
			Scalar (intercepts)	0.869	0.859	0.077	0.071	−0.034	0.009	0.004	Not invariant

For the four-group CFA, partial scalar invariance was examined by sequentially freeing intercept constraints guided by modification indices while retaining at least two invariant intercepts per factor. Even after freeing 9 intercept constraints, the partial scalar model did not satisfy the prespecified ΔCFI criterion when compared with the metric invariance model.

Decision criteria: ΔCFI ≤ 0.010, ΔRMSEA ≤ 0.015; ΔSRMR ≤ 0.030 (metric) / ≤ 0.015 (scalar).

Supplementary Table S5 shows the average subscale scores (descriptive) of MCCQ-J for each caffeinated beverage under beverage-specific framing. Omnibus ANOVA results survived BH-FDR correction for all subscales except symptom management (*q* < 0.05), with coffee having the highest scores for all subscales; however, these observed mean comparisons should be treated as descriptive and exploratory rather than as measurement-equivalent group comparisons given the lack of full scalar invariance and acceptable partial scalar invariance across beverage types. Differences between *ryoku-cha* (green tea) and *kou-cha* (black tea) were small across the six subscales and are reported for descriptive purposes.

## 4 Discussion

The current study had two aims: (1) to examine whether the suboptimal fit under all-beverage framing was consistent with a *ryoku-cha* (green tea)-specific context explanation by comparing all-beverage and beverage-specific framings and evaluating measurement invariance; and (2) to descriptively explore beverage-wise score patterns among Japanese participants under the original six-factor MCCQ framework.

Regarding aim (1), several findings suggested that the suboptimal fit of the six-factor model under all-beverage framing is unlikely to be explained solely by a green-tea-specific context. To better understand the observed misfit, we consider possible explanations related to beverage context, item- and structure-related characteristics of the MCCQ-J, and participant characteristics.

First, several findings indicated that the observed model misfit was not specific to a green-tea context but may instead reflect factors associated with other beverage contexts as well.

In beverage-specific two-group CFAs, scalar invariance was not consistently determined by whether a pair included green tea: scalar invariance failed for some beverage pairs that did not include green tea, while it held for some pairs that did include green tea (Table 7). These results are inconsistent with a green-tea-specific explanation of measurement non-invariance across beverage types. At the same time, scalar invariance for the coffee vs. *ryoku*-*cha* pair may indicate some similarity between these two beverage contexts, rather than ruling out any role of green tea in response heterogeneity. This pattern does not rule out the possibility that green tea contributes to response heterogeneity in some respects, but it suggests that green tea alone is unlikely to explain the observed misfit of the six-factor MCCQ-J model.

Similarly, under beverage-specific framing, CFAs for each beverage type showed that model fit was not uniformly improved by specifying a single beverage. Notably, the coffee condition exhibited relatively poorer fit than the green tea condition (Table 3). This result further weakens the interpretation that only green tea is the primary cause of misfit.

Furthermore, approximately 80% of participants reported coffee as the beverage that first comes to mind when thinking about caffeinated beverages (Table 1). This result is consistent with a previous study suggesting that Japanese university student's most strongly associate caffeine with coffee compared with other beverages (12). Additionally, except for symptom management, correlations between the same subscale scores obtained under all-beverage framing and under beverage-specific framing tended to be strongest among participants assigned to the coffee condition (Supplementary Table S10). These findings suggest that many participants may have primarily had coffee in mind even when responding under the all-beverage framing. Taken together, these findings suggest that the observed model misfit cannot be attributed solely to a uniquely green-tea-specific context.

Second, several findings suggested that the observed model misfit may partly reflect item- and structure-related characteristics of the MCCQ-J itself rather than beverage context alone.

Item-level misfit under all-beverage framing (Supplementary Table S6) further suggested that model misfit was not limited to a specific beverage context but was partly associated with particular questionnaire items. Notably, several alertness-related items showed large modification indices or standardized residual covariances, and one alertness item (“because I become refreshed”) showed a potential cross-loading on the taste factor. These findings suggest that some alertness-related items may require further examination in Japanese and support the need for additional refinement of the MCCQ-J rather than a beverage-specific explanation alone. For example, Japanese expressions corresponding to being “refreshed” may overlap with feelings of sensory enjoyment or taste-related impressions, which could partly explain the potential cross-loading of item 13 on the taste factor. However, this interpretation remains tentative and should be examined through cognitive interviews.

Furthermore, the consistently suboptimal fit observed across all beverage conditions also raises the possibility that the original MCCQ structure may not function optimally in Japanese samples. However, previous exploratory analyses of a younger Japanese sample did not identify a clear alternative factor structure that substantially improved upon the provisional six-factor framework (5). Therefore, the present findings do not provide a clear basis for replacing the original factor structure. Nevertheless, further research may be needed to examine whether alternative modeling approaches (e.g., ESEM) or culturally adapted item wording can improve the psychometric performance of the MCCQ-J in Japanese samples.

Finally, model fit varied modestly across demographic groups: relative fit tended to be poorer among females and among participants in their 50s (Table 2). Although these differences were not large, they suggest that sample composition could slightly amplify model misfit. In the current sample, the proportion of female participants was lower than in the original Hungarian sample, but the mean age was higher. Therefore, age differences in the motives or response styles might contribute to MCCQ-J's misfit.

Regarding aim (2), the four-group measurement invariance analysis under beverage-specific framing indicated that scalar or acceptable partial scalar invariance across beverage types was not established. Therefore, beverage-type differences in motive scores could not be interpreted as measurement-equivalent latent mean differences (Table 7). At a descriptive level, observed subscale means under beverage-specific framing varied across beverage types, with coffee tending to show higher observed scores than the other beverages across several motives (Supplementary Table S5). Because assigned beverage groups differed by gender and age group, these descriptive differences may also partly reflect demographic composition rather than beverage context alone. These patterns should be regarded as exploratory descriptive findings rather than evidence of robust between-beverage differences. Separately, because cross-national measurement invariance cannot be tested with the current data, the data cannot support substantive cultural differences in motive levels or rank orders. Given this limitation, the observed rank-order pattern under all-beverage framing appeared broadly similar to that reported in the original MCCQ study, although mood motives were descriptively more prominent relative to alertness in the Japanese sample [see Table 5; see also Ágoston et al. (2)]. These observations should be treated as tentative rather than definitive evidence.

## 5 Limitations

First, the MCCQ-J was provisionally adapted via forward-backward translation, and evidence for conceptual equivalence remains limited. Therefore, culture-specific nuances in wording and interpretation may remain and could have affected model fit and score comparisons.

Second, the sample was recruited from an online survey panel and excluded individuals who themselves or whose cohabiting family members worked in the food and beverage, food service, mass media, or marketing research industries. The use of an online panel and these exclusions may also introduce sampling bias and limit generalizability to Japanese caffeine consumers (e.g., underrepresenting individuals with higher domain knowledge). Furthermore, the study was limited to adults aged 20–59 years. Because beverage consumption patterns, including the relative importance of tea, may vary across age groups, the findings may not generalize to adults aged 60 years or older.

Third, because attrition from Survey 1 to Survey 2 was associated with age group, the age composition of the follow-up analytic sample may have influenced the beverage-specific CFA and measurement invariance results. Although age-group invariance was supported under all-beverage framing and baseline behavioral and MCCQ-J variables showed limited evidence of attrition bias, the possibility that age-related response tendencies affected model fit under beverage-specific framing cannot be ruled out.

Fourth, because beverage assignment was constrained by participants' reported consumption of the four target beverages, assigned beverage groups differed in gender and age composition. Therefore, descriptive beverage-type differences may partly reflect participant characteristics associated with beverage choice rather than beverage context alone. In addition, some MCCQ-J subscale scores differed by gender and showed associations with age in the present sample. Consequently, observed differences in CFA fit (including the relatively better fit observed in the energy drink condition) or measurement-invariance results across beverage conditions may also partly reflect demographic differences between beverage groups rather than beverage context alone. Beverage groups may also have differed in unmeasured characteristics, such as personality traits, lifestyle, habitual caffeine use, caffeine dependence, or culturally shaped beverage practices.

Fifth, although positive associations between age and some motive scores (e.g., habit and taste) were observed, because the data were cross-sectional, it is unclear whether these associations reflect age-related changes or generational differences.

Sixth, because previous studies have reported an association between coffee consumption motives and personality-related variables (13), future studies should examine whether personality traits contribute to individual differences in MCCQ-J response patterns. However, the present study did not measure personality traits in Survey 2.

Seventh, scalar or acceptable partial scalar invariance across beverage types was not established. Therefore, beverage-type differences could not be evaluated through measurement-equivalent latent mean comparisons in this study, and the observed mean patterns should be interpreted only descriptively.

Finally, it is unclear how the results of this study would be affected if we took into account beverage temperature. For example, motives for consuming hot coffee and iced coffee may differ.

## 6 Conclusion

This study preliminarily examined Japanese motive-related score patterns for consuming caffeinated beverages under the provisional six-factor MCCQ-J framework. Overall, the six-factor solution showed suboptimal fit under both all-beverage and beverage-specific framings, indicating that evidence for the structural validity and beverage-type comparability of this framework in Japan remains limited. Moreover, the pattern of model fit and measurement non-invariance was inconsistent with a green-tea-specific explanation for the MCCQ-J misfit. In addition, Japanese participants descriptively tended to report higher mood motive-related scores than those reported in the original MCCQ study; however, this observation should be interpreted cautiously because cross-national measurement invariance was not tested. Future work should refine the MCCQ-J through cognitive interviews with Japanese samples, possible item rewording, and pilot testing, before strong beverage-type or cross-national comparisons are attempted. Despite these limitations, this study provides preliminary reference points on Japanese motive profiles for caffeinated beverage consumption.

## Data Availability

The raw data supporting the conclusions of this article will be made available by the authors, without undue reservation.
